# Polymeric Nanoparticles Enable Targeted Visualization of Drug Delivery in Breast Cancer

**DOI:** 10.1021/acs.molpharmaceut.4c00695

**Published:** 2025-04-21

**Authors:** Md. Jashim Uddin, Justin Han-Je Lo, Mukesh K. Gupta, Thomas A. Werfel, Abu Asaduzzaman, Connor G. Oltman, Eva F. Gbur, Mohammed T. Mohyuddin, Farhana Nazmin, Md. Saidur Rahman, Ahan Jashim, Brenda C. Crews, Philip J. Kingsley, Jamie E. Klendworth, Lawrence J. Marnett, Craig L. Duvall, Rebecca S. Cook

**Affiliations:** † Department of Biochemistry, Vanderbilt University School of Medicine, Nashville, Tennessee 37232, United States; ‡ Department of Biomedical Engineering, Vanderbilt University School of Engineering, Nashville, Tennessee 37232, United States; § Departments of Electrical and Computer Engineering, Wichita State University School of Engineering, Wichita, Kansas 67260, United States; ∥ Department of Medicine, Vanderbilt University Medical Center, Nashville, Tennessee 37232, United States; ⊥ Departments of Biochemistry, Chemistry and Pharmacology, Vanderbilt University School of Medicine, Nashville, Tennessee 37232, United States

**Keywords:** fluorocoxib Q, chemocoxib A, micellar nanoparticles, tumor imaging, drug delivery

## Abstract

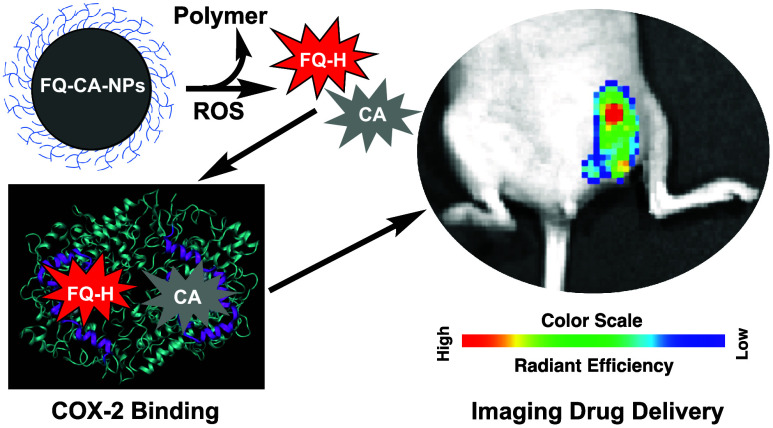

We report the coencapsulation of fluorocoxib Q (FQ) and chemocoxib A (CA) in micellar nanoparticles (FQ-CA-NPs) of a new PPS_135_-*b*-POEGA_17_ diblock polymer, which exhibited a hydrodynamic diameter of 109.2 ± 4.1 nm and a zeta potential (ζ) of −1.59 ± 0.3 mV. The uptake of FQ-CA-NPs by 4T1 mouse mammary cancer cells and intracellular cargo release were assessed by fluorescence microscopy that resulted in increased fluorescence in 4T1 cells compared to cells pretreated with celecoxib. The viability of primary human mammary epithelial cells (HMECs) or 4T1 mouse mammary carcinoma cells treated with FQ-CA-NPs were assessed, which showed decreased growth of 4T1 breast cancer cells but showed no effect on the growth of primary human mammary epithelial cells (HMECs). Intravenous dosing of FQ-CA-NPs in mice enabled ROS-induced cargo (FQ and CA) release and fluorescence activation of FQ and resulted in increased fluorescence in breast tumors compared to the tumors of animals pretreated with tempol or celecoxib, and minimum fluorescence was detected in the tumors of animals treated with nothing or empty-NPs. In addition, tumor tissues from treated animals were analyzed *ex vivo* by liquid chromatography–mass spectrometry (LC–MS)/MS, and identified increased levels of cargo delivery and retention in the tumor compared to tempol- or celecoxib-pretreated animal tumors. These *in vivo* and *ex vivo* results confirmed the targeted delivery of loaded NPs followed by ROS-mediated cargo release and fluorescence activation for targeted visualization of drug delivery in breast tumors and CA-induced therapeutic effect in an *in vivo* tumor growth inhibition assay and an *ex vivo* hematoxylin and eosin (H&E) staining of tumor tissues. Thus, coencapsulation of FQ and CA into polymeric micellar nanoparticles (FQ-CA-NPs) enabled their ROS-sensitive release followed by fluorescence activation and COX-2-dependent tumor targeting and retention in the visualization of CA delivery in solid breast tumors.

## Introduction

Nanomedicine formulations of systemically dosed chemotherapeutic drugs aim to improve the biodistribution and target-site accumulation of cytotoxic drugs.^1^ Various passively and actively targeted nanomedicines have been evaluated, e.g., liposomes,^2^ polymer micelles,^3,4^ and antibodies.^5^ Previous studies showed that 5–200 nm-sized nanoparticles can improve the therapeutic index of low molecular weight drugs.^1,2,6−8^ Nanomedicine formulations can also be used in imaging applications.^4,9,10^ Personalized medicine will benefit from a new paradigm that enables codelivery of imaging and therapeutic agents into cancers for imaging during treatment, complementing current tumor imaging studies that occur only before and after treatment.

Studies have shown that cyclooxygenase-2 (COX-2) contributes to several pathologies, including cancer.^11,12^ Importantly, COX-2 and reactive oxygen species (ROS) colocalize in pathologic tissues.^13^ COX-2 is an attractive molecular target for delivering imaging/therapeutic agents to tumors because it is expressed in only a few normal tissues and is greatly up-regulated in inflamed tissues and many premalignant and malignant tumors.^11,14,15^ We reported the synthesis and *in vivo* validation of radiologic imaging agents for both single photon emission computed tomography (SPECT) and positron emission tomography (PET) imaging. The structure of these agents is based on the indomethacin and celecoxib scaffolds.^16,17^ We validated the specificity and sensitivity of our radiolabeled probes in COX-2-targeted imaging of inflammation and cancer both *in vitro* and *in vivo*, and e*x vivo*.^16,17^ In addition, fluorescent COX-2 inhibitors are attractive candidates as targeted optical imaging agents. Such compounds have the advantage that each molecule bears the fluorescence labeling, and the compounds are nonradioactive and stable.^9,11,18,19^ Thus, they can be used for cellular imaging, animal imaging, and clinical imaging of tissues where topical or endoluminal illumination is possible (e.g., esophagus, colon, and upper airway via endoscopy, colonoscopy, and bronchoscopy, respectively).^15^ Prior work from our laboratory demonstrated that fluorescent COX-2 inhibitors are useful as chemical probes for protein binding and *in vivo* imaging.^11,13,14,18−21^ We designed and synthesized COX-2-targeted imaging agents, and later, we successfully discovered a novel redox-activatable COX-2-targeted optical imaging agent, called fluorocoxib Q (FQ).^13^ Fluorocoxib Q is a nitroxide derivative of fluorocoxib A. Fluorocoxib Q enables targeted visualization of COX-2 and ROS in pathological tissues.^13^ Fluorocoxib Q exhibits extremely low fluorescence emission due to the quenching of the excited electronic state of the carboxy-X-rhodamine by the nitroxide radical within the molecule.^13^ Upon radical trapping in cancer cells by reactive oxygen species (ROS), the COX-2-targeted fluorocoxib Q probe becomes fluorescently activated, making it effective for tumor imaging.

Overexpression of COX-2 and its prostaglandin products play a vital role in tumorigenesis.^22^ Our prior studies demonstrate COX-2-targeted delivery of imaging agents to tumors,^9,11,14,18,20,21,23−25^ suggesting the possibility that this approach can be used to selectively deliver chemotherapeutic agents as well. In an attempt to test this hypothesis, we used a conjugate chemistry approach, where we discovered a cytotoxic COX-2 inhibitor, called chemocoxib A (CA), a conjugate of indomethacin and podophyllotoxin, which revealed highly potent and selective COX-2 inhibition in purified enzyme and in cell-based assays.^26^ X-ray cocrystallographic studies demonstrated the structural basis of COX-2 binding by CA, and kinetic analysis demonstrated that CA is a slow, tight-binding inhibitor of COX-2. The conjugate exhibited cytotoxicity in cell cultures similar to that of podophyllotoxin. It was accumulated selectively in COX-2 positive tumors in mice and induced tumor growth inhibition by >50% but did not have any adverse effect on tumors that did not express COX-2 enzyme.^26^ It should be noted that podophyllotoxin itself does not inhibit COX-2, but when tethered to indomethacin, it does and accumulates in COX-2-expressing tumor cells. How CA slows the growth of cancer cells *in vitro* and *in vivo* is not fully understood, but it does not affect the viability of primary human mammary epithelial cells (HMECs) in culture.^26^ Also, it does not cause systemic toxicity in animals.^26^ Thus, chemocoxib A provides a proof-of-concept for *in vivo* targeting of chemotherapeutic agents to COX-2 and represents the first cytotoxic COX-2 inhibitor validated for targeted tumor growth inhibition *in vivo*. We hypothesize that fluorocoxib Q^13^ and chemocoxib A^26^ can be coencapsulated into ROS-responsive polymeric micellar nanoparticles that can codeliver both agents into tumors to allow image-based confirmation of drug delivery into solid tumors with molecular specificity in real time. Herein, we report the discovery of FQ- and CA-coloaded PPS_135_-*b*-POEGA_17_ polymeric micellar nanoparticles (FQ-CA-NPs) and their *in vivo* delivery into COX-2-expressing orthotopic mouse mammary tumors.

## Materials and Methods

Fluorocoxib Q (FQ), chemocoxib A, and the diblock PPS_135_-*b*-POEGA_17_ copolymer were synthesized using previously published procedures (see Supporting Information).^9,13,26,27^

### Coencapsulation of FQ and CA in PS_135_-*b*-POEGA_17_ Polymeric Micellar Nanoparticles

FQ and CA were coencapsulated into PPS_135_-*b*-POEGA_17_ copolymeric micelles using a bulk solvent evaporation method.^9^ First, FQ, CA, and PPS_135_-*b*-POEGA_17_ were each dissolved in chloroform separately. FQ (10 mL, 20 mg/mL) and CA (10 mL, 20 mg/mL) solutions were added to the PPS_135_-*b*-POEGA_17_ copolymer (20 mL, 200 mg/mL) solution. The resultant solution was added dropwise to Dulbecco’s phosphate-buffered saline (1 mL, DPBS, pH 7.0–7.3, Ca^2+^ and Mg^2+^-free) followed by 16 h gentle stirring at 25 °C in the dark. Sterile centrifugation of the resultant aqueous solution afforded FQ- and CA-coloaded nanoparticles (FQ-CA-NPs) ready for *in vitro* and *in vivo* evaluations.

### Characterization of NPs

A Malvern Zetasizer Nano-ZS Instrument equipped with a 4 mW helium–neon laser operating at 632.8 nm was used to measure the hydrodynamic diameter (*D*_h_) and zeta potential (ζ) of the micellar FQ-CA-NPs. Each dissolved in DPBS was passed through a 0.45 μm syringe filter. Transmission electron microscopy (TEM) samples of empty-NPs or FQ-CA-NPs were developed by adding 5 μL of NP solution to pure carbon TEM grids and counterstaining with uranyl acetate (3% solution, 5 μL). The grids were then dried overnight under vacuum and imaged by TEM on a FEI Tecnai Osiris microscope operating at 200 kV.

### Concentration of FQ and CA in Micellar Nanoparticles

The concentration of FQ and CA within micellar nanoparticles (FQ-CA-NPs, 0.5 mL in DPBS) was measured by first disassembling NPs in dimethylformamide (DMF, 0.5 mL, stirring 16 h, at 25 °C). Samples were dried, reconstituted, and analyzed by reversed-phase HPLC-UV using Phenomenex C18 columns held at 40 °C. Each compound was quantified against a standard curve obtained after dissolving it in 50% DMF in DPBS. The statistical comparisons of the experimental results were performed by Student’s *t*-test at significance levels of 0.01 and 0.001, which afforded FQ and CA concentrations of 0.132 and 0.147 mg/mL in a typical FQ-CA-NPs injectable formulation.

### H_2_O_2_-Dependent Cargo Release

FQ-CA-NPs were incubated in H_2_O_2_ (ROS) at progressively increasing concentrations (0–1000 mM) in 96-well plates. Fluorescence intensity was monitored using a Tecan Infinite 500 plate reader (excitation 540 nm, emission 610 nm), normalizing each sample to its fluorescence value prior to the addition of H_2_O_2_.

### Cells

Primary human mammary epithelial cells (HMECs) were obtained as a gift from Dr. Jennifer Pietenpol, Vanderbilt University Medical Center, and mouse 4T1 mammary carcinoma cells were obtained from the Cell Culture and Tissue Engineering Core of the Vanderbilt Institute for Integrative Biosystems Research and Education.

### Fluorescence Microscopy of 4T1 Cells

Mouse 4T1 mammary carcinoma tumor cells were obtained from the Cell Culture and Tissue Engineering Core of the Vanderbilt Institute for Integrative Biosystems Research and Education. The 4T1 cells (1.6 × 10^5^ cells) were plated on MatTek glass-bottom culture dishes. The 4T1 cells in Hank’s balanced salt solution (HBSS)/Tyrode’s were grown to 60% confluency and incubated with 200 nM of fluorocoxib Q for 3 h at 37 °C. Adherent cells were treated with FQ-CA-NPs (1 μM). Cells were rinsed 3 times with Hanks balanced salt solution (HBSS)/Tyrode’s buffer and imaged at a gain of 20 on a Leica DM IL LED FIM fluorescence microscope.

### Cytotoxicity Assays

The primary human mammary epithelial cells (HMECs) were grown in HMEC medium containing mammary epithelial growth supplement (Life Technologies) or mouse 4T1 mammary carcinoma cells were grown in growth media (Dulbecco’s modified Eagle’s medium:nutrient mixture F12 plus 10% fetal bovine serum and antibiotic/antimycotic). Cells (8000 to 10,000/well) were plated in 96-well plates (Sarstedt). After 24 h, the cells were treated with FQ-CA-NPs (ca. 200 nmol of CA) or vehicle in fresh growth media. Viability was assessed 48 h later using WST-1 Cell Proliferation Reagent (Roche, 11644807001), as described previously.^28^

### In Vivo Pharmacokinetic Analysis of FQ and CA

The animal experiments were performed using the Vanderbilt Institutional Animal Care and Use Committee (IACUC) approved protocols. To determine pharmacokinetic parameters, we dosed a group of C57BL/6 mice with FQ (10 mg/kg) and a second group of C57BL/6 mice with CA (10 mg/kg) by intravenous injections. We collected blood samples via cardiac puncture at 0, 3, 12, 24, and 48 h postinjection (*n* = 4 animals at each time point). We centrifuged the collected blood, obtained plasma samples from both groups, and stored the isolated plasma samples at −80 °C until analysis. We quantified the levels of FQ and CA in the plasma by an acetonitrile-freeze method and analyzed them by an LC–MS/MS analysis method.

### Animal Model of Orthotopic Breast Cancer

We developed an animal model of orthotopic breast cancer in NU/J mice using a protocol approved by the Institutional Animal Care and Use Committee (IACUC) at the Vanderbilt University School of Medicine. We used female homozygous NU/J mice (6 weeks) that were purchased from the Jackson Laboratory, Sacramento. As indicated in individual studies, we injected either 1 million 4T1 (mouse) or MDA-MB-231 (human) triple-negative breast cancer cells into the left mammary fat pad. For fluorescence imaging studies, the injected animals were housed with a normal sterile diet for 1–2 weeks. During this growth period, the body weight was measured by an animal weighing balance (CJ-4000 Kent Scientific), and tumor volume was determined every day using a digital Vernier caliper to allow the breast tumor to grow up to 700–900 mm^3^.

### Fluorescence Imaging of Drug Delivery in a Mouse Model of Orthotopic Breast Cancer

All experiments involving mice were approved by the Institutional Animal Care and Use Committee (IACUC) at the Vanderbilt University School of Medicine. The FQ-CA-NPs (1 mg/kg of each CA and FQ) or empty-NPs were dosed by subcutaneous injection to NU/J mice harboring orthotopic breast tumors (4 animals/group). In an additional control experiment, tempol (10 mg/kg) or celecoxib (10 mg/kg) was injected intraperitoneally for 1 h before subcutaneous injection of FQ-CA-NPs (1 mg/kg of each CA and FQ) to NU/J mice harboring orthotopic breast tumors (4 animals/group). At 49 h postinjection of loaded or empty-NPs, animals were lightly anesthetized with 2% isoflurane and imaged using a Xenogen IVIS 200 Optical Imaging system (DsRed filter spanning 570–615 nm with a 20 cm field of view at 20 μm resolution at a depth of 1.5 cm, and an exposure of 1 s). The static images were analyzed using ImageJ software. After *in vivo* imaging, mice were humanely euthanized, and the tumor, brain, liver, lung, and kidney were collected. The dissected organs were imaged *ex vivo* on a Xenogen IVIS 200 Optical Imaging Instrument. Using ImageJ software, the images were analyzed, and regions of interest (ROIs) were created for measurements.

### Treatment of Orthotopic Breast Tumor Models and Histological Analysis

Orthotopic mammary fat pad tumor xenografts (MDA-MB-231) were implanted in nude mice as detailed above and allowed to engraft for 3 weeks and grow to a volume of approximately 100 mm^3^. Mice were then treated with 1 mg/kg intravenously (by loaded drug equivalent) of FQ-CA-NPs or empty (control) NPs twice weekly for a total of 4 doses, with mice sacrificed 18 days after the start of treatment. Tumor size was measured noninvasively using calipers, with tumor volume estimated as an ellipsoid with volume (π/6) × *a* × *b*^2^, where *a* is the diameter of the major axis (length) and *b* is the diameter of the minor axis (width and depth). Tumors, livers, spleens, and kidneys were taken during necropsy and fixed in 10% neutral buffered formalin for 1 week, then processed/embedded in paraffin at the Vanderbilt Translational Pathology Shared Resource (TPSR), sectioned (Epredia HM 325), and stained with hematoxylin (Fisher) and eosin Y (Abcam) using standard procedures. Slides were scanned using an Epredia Pannoramic MIDI II and analyzed in QuPath v0.5.1.

### Tissue Analysis

After *in vivo* imaging of the animals, followed by *ex vivo* imaging of major organs, including tumors, they were stored at −50 °C until analysis. All of the tumors (4 tumors/group) in each group (test or control) were combined, weighed, and homogenized in 1:1 methanol:DPBS. The homogenate was stored at −20 °C overnight and centrifuged for 10 min at 4 °C, and 75% of the clear supernatant was partially dried under nitrogen to reduce the volume and remove methanol. To the partially dried sample, a volume of 1% acetic acid (roughly 5× the amount of homogenate remaining) was added, and the sample was loaded onto a conditioned Phenomenex C18 solid-phase extraction cartridge. After loading, the cartridge was rinsed with 5 mL of 1% acetic acid and then with 5 mL of 1% acetic acid containing 14% methanol. The eluent was dried under nitrogen and reconstituted in 100 μL methanol followed by 100 μL water. The reconstituted samples were analyzed via liquid chromatography–mass spectrometry (LC–MS)/MS.

### Statistical Methods and Analysis

Student’s *t*-test was used for statistical analyses of fluorescence signal intensities in static images of breast tumors or major organs, including the lung, liver, kidney, or brain, where statistical significance at *P* ≤ 0.05 was considered. Within the size of the samples (*n*), the signal intensities were used as the arithmetic mean and standard error.

## Results and Discussion

We synthesized fluorocoxib Q (FQ),^13^ a nitroxide analog of fluorocoxib A.^11^ The chemical synthesis and spectroscopic characterization of FQ are described in the Supporting Information section (Scheme S1). FQ is pro-fluorescent, which is due to quenching of its carboxy-X-rhodamine fluorescence by the nitroxide radical within the molecule and is a redox-activatable COX-2-targeted imaging agent exhibiting extremely low fluorescence emission until activated by ROS present in pathologic tissues.^13^ Next, we synthesized chemocoxib A (CA),^4^ a cytotoxic COX-2 inhibitor exhibiting potent antitumor activity.^26^ The chemical synthesis and spectroscopic characterization of CA are described in the Supporting Information section (Scheme S2). CA is an indomethacin–podophyllotoxin conjugate that demonstrated tumor growth inhibition without any systemic toxicity.^26^

**Figure 1:**
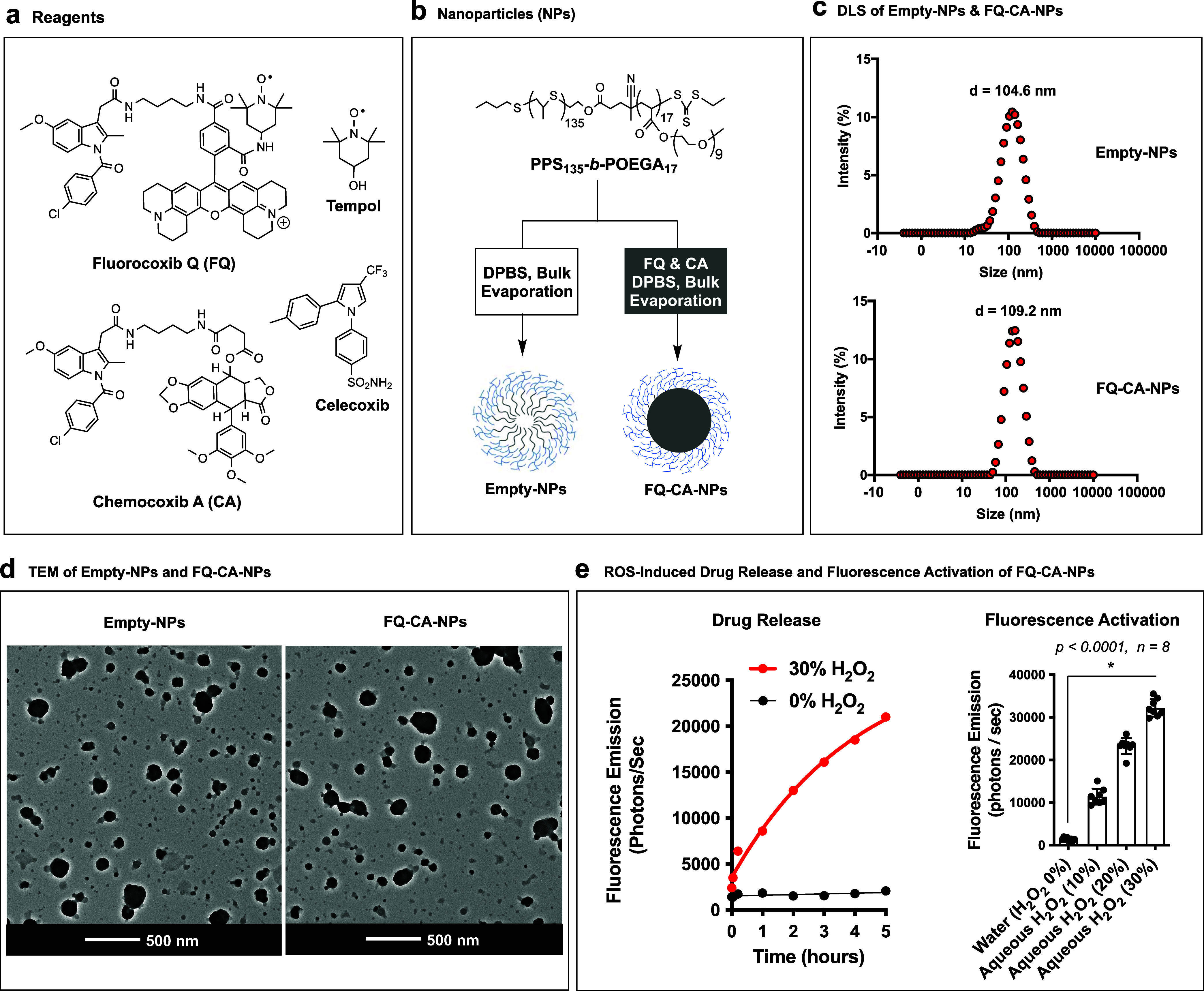
(a) Chemical structures of fluorocoxib Q (FQ), chemocoxib A (CA), Tempol, and celecoxib. (b) Formulation of empty-NPs and coencapsulation of FQ and CA using a bulk evaporation method for the formation of micellar nanoparticles (FQ-CA-NPs) with PPS_135_-*b*-POEGA_17_ polymer in a 50:50 mixture of CHCl_3_/DPBS (v/v) with gentle stirring at 25 °C for 16 h. (c) Dynamic light scattering of empty-NPs and FQ-CA-NPs. (d) Transmission electron microscopy (TEM) of empty-NPs and FQ-CA-NPs. (e) ROS-induced drug release and fluorescence activation of FQ-CA-NPs treated with aqueous hydrogen peroxide (H_2_O_2_) solutions.

In addition, we synthesized PPS_135_-*b*-POEGA_17_, an amphiphilic diblock copolymer that self-assembles into micellar nanoparticles <200 nm in hydrodynamic diameter. The new amphiphilic diblock copolymer, PPS_135_-*b*-POEGA_17_, is a structural and functional derivative of the previously developed and reported copolymer PPS_106_-*b*-POEGA_17_.^9^ The chemical synthesis and spectroscopic characterization of PPS_135_-*b*-POEGA_17_ are described in the Supporting Information section (Scheme S3). This new copolymer was developed to enable the coencapsulation of FQ and CA. This derivatization was necessary because the coencapsulation of FQ and CA with PPS_106_-*b*-POEGA_17_ was unsuccessful. We coencapsulated FQ and CA into ROS-responsive micellar nanoparticles (FQ-CA-NPs) with PPS_135_-*b*-POEGA_17_ using a bulk solvent evaporation method (Figure 1).^9^ We selected this DP of individual block for drug-loadable PPS135-*b*-POEGA17 considering the 1:1 ratio of hydrophobic to hydrophilic polymer in the diblock copolymer, which favors the formation of micellar nanoparticles over other structures in amphiphilic diblock copolymers.^29^ PPS135-*b*-OPEGA17 consists of 135 hydrophobic PS units and 153 hydrophilic ethylene glycol (EG) units (17 × 9 = 153, 9 EG units from each OPEGA unit), which formed stable nanoparticles in aqueous solution, confirmed by DLS-based size measurement and TEM imaging.^9^ We have successfully used this length of hydrophobic PPS polymers in other drug-loadable nanoparticle formulations.^9,30,31^ Increasing the percentage of hydrophobic block ratio over hydrophilic block improves micelles’ drug-loading capability.^32^ However, a higher ratio of hydrophobic blocks also reduces the stability of the micellar nanoparticles. Therefore, the combined DPs of PPS (135 PS units) and OPEGA (17 units) in the amphiphilic diblock copolymer provided the optimum balance for micellar nanoparticle stability and drug loading.

In this method, FQ, CA, and PPS_135_-*b*-POEGA_17_ were dissolved in chloroform singly. Then, FQ and CA solutions were mixed and the mixture was added to the PPS_135_-*b*-POEGA_17_ copolymer solution. The resultant solution was then added dropwise to 1 mL of Dulbecco’s phosphate-buffered saline (DPBS pH 7.0–7.3, without calcium and magnesium) at 25 °C with gentle stirring. The binary system was stirred for 16 h at 25 °C in the dark, during which time chloroform was evaporated. Sterile centrifugation of the formulation afforded FQ- and CA-coloaded micellar nanoparticles (FQ-CA-NPs) ready to be used for evaluation and visualization of cells and CA delivery in orthotopic mammary tumors implanted in nude mice.

We determined the hydrodynamic diameter of empty-NPs to be 104.6 ± 3.7 nm and that of FQ-CA-NPs to be 109.2 ± 4.1 nm. In addition, we determined the zeta potential (ζ) of empty-NPs to be −1.38 ± 0.7 mV and that of FQ-CA-NPs to be −1.59 ± 0.3 mV. Further, we acquired the transmission electron microscopy (TEM) images of empty-NPs or FQ-CA-NPs, as shown in Figure 1d. Then, we treated FQ-CA-NPs with H_2_O_2_ solutions at a range concentration that showed higher fluorescence with 30% as compared to 20% or lower H_2_O_2_ concentration in water, suggesting its sensitivity to ROS allowing drug release and fluorescence activation of FQ.

**Figure 2:**
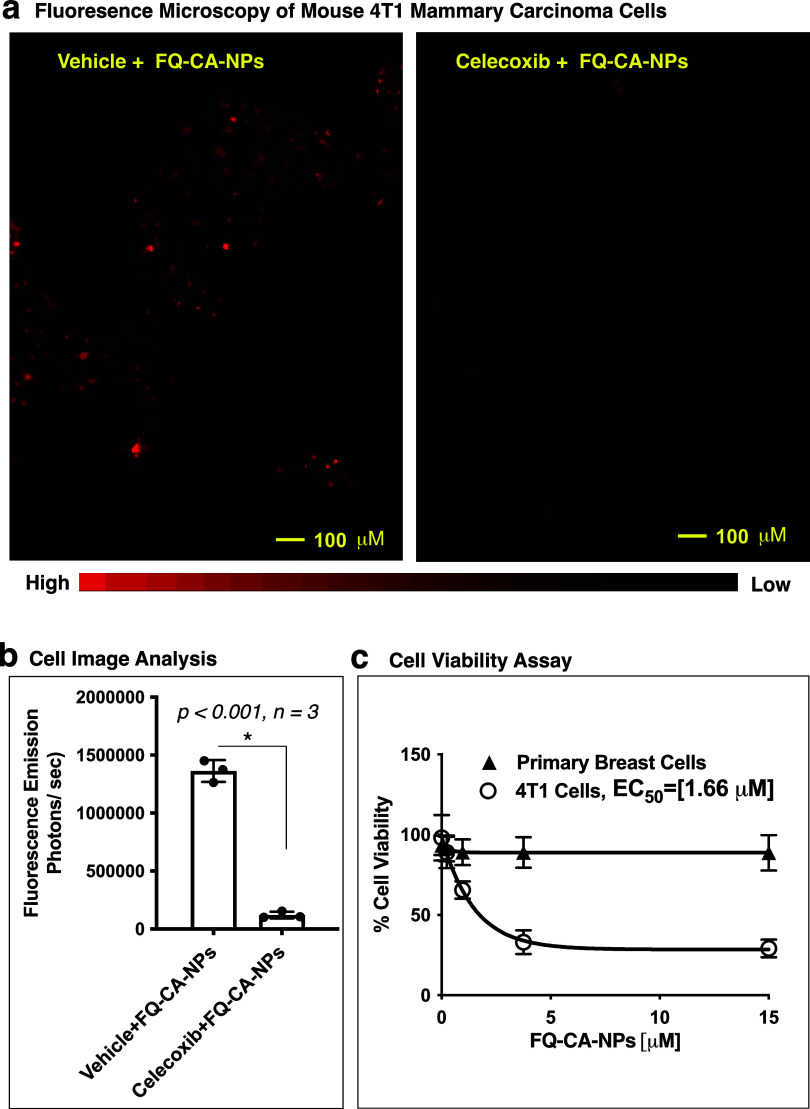
(a) Fluorescence microscopy of vehicle or celecoxib-pretreated 4T1 cells incubated with FQ-CA-NPs for 3 h. (b) Image analysis of vehicle or celecoxib-pretreated 4T1 cells incubated with FQ-CA-NPs using ImageJ software. (c) Viability of primary human mammary epithelial cells (HMECs) or 4T1 mouse mammary carcinoma cells treated with FQ-CA-NPs for 48 h.

We evaluated the coloaded nanoparticles in optical imaging of cells, where FQ-CA-NPs showed higher fluorescence in vehicle-pretreated 4T1 cells as compared to celecoxib-pretreated 4T1 cells with a significant difference in light emission (Figure 2a,b). In a cell viability assay, the FQ-CA-NPs were toxic to 4T1 breast carcinoma cells with an EC_50_ of 1.66 μM, but nontoxic to primary human mammary epithelial cells (HMECs) (Figure 2c).

**Figure 3:**
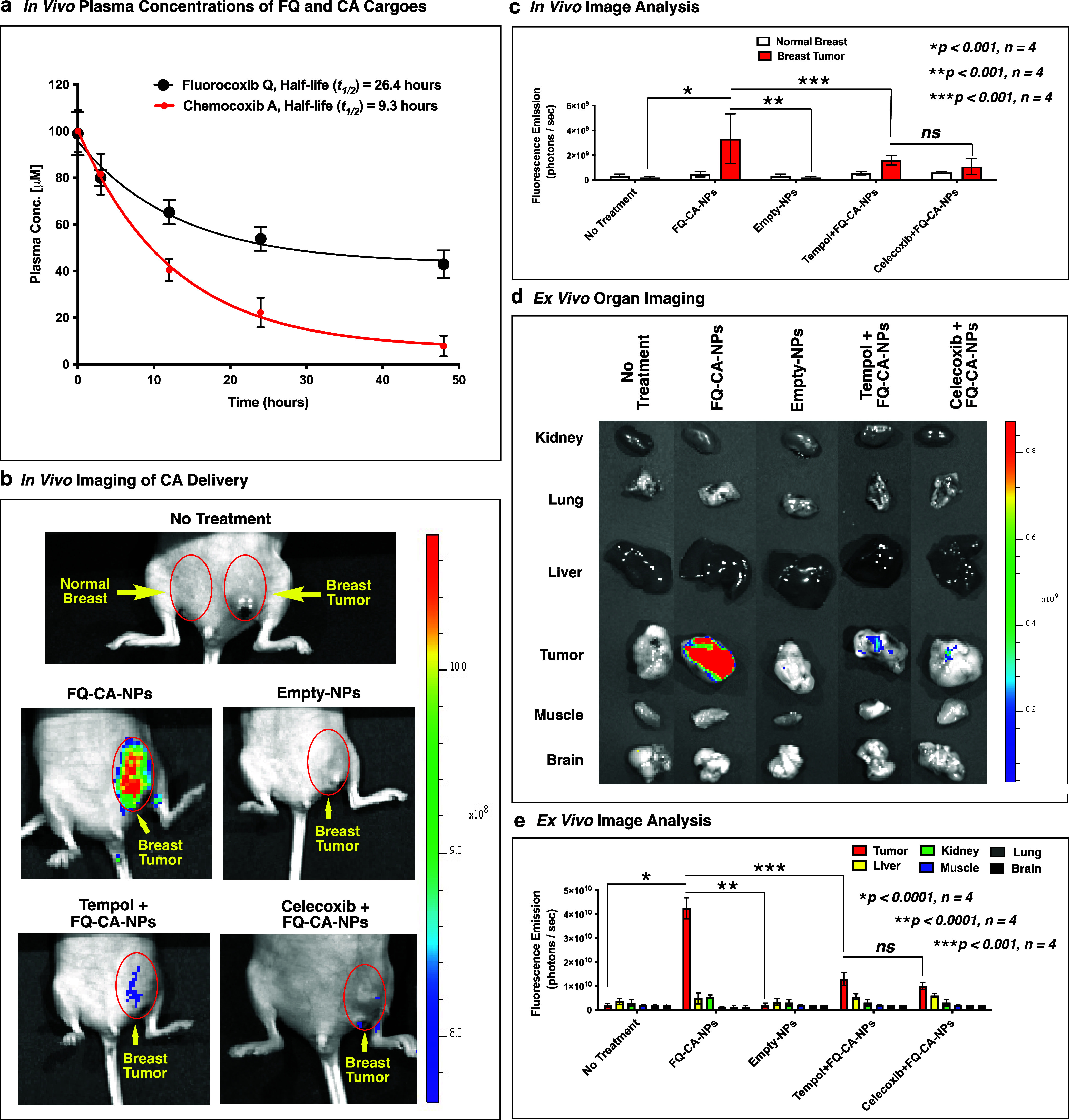
(a) *In vivo* pharmacokinetics of FQ and CA in C57BL/6 mice. (b) *In vivo* optical imaging of NU/J mice bearing orthotopic 4T1 mammary tumors at 49 h after intravenous administration of FQ-CA-NPs, empty-NPs, and tempol followed by FQ-CA-NPs, and celecoxib followed by FQ-CA-NPs. (c) Measurement of light emission in the normal breast and breast tumors by ImageJ software. (d) *Ex vivo* optical imaging of the kidney, lung, liver, tumor, muscle, and brain of NU/J mice bearing orthotopic 4T1 mammary tumors at 49 h after intravenous administration of FQ-CA-NPs, empty-NPs, and tempol followed by FQ-CA-NPs, and celecoxib followed by FQ-CA-NPs. (e) Measurement of light emission in the excised kidney, lung, liver, tumor, muscle, and brain by ImageJ software.

We determined the *in vivo* pharmacokinetics of FQ and CA in C57BL/6 mice. In this assay, FQ resulted in a plasma half-life of 26.4 h and CA showed 9.3 h (Figure 3a). We dosed FQ-CA-NPs by subcutaneous injection to mice harboring orthotopic mouse mammary tumors (4T1) expressing COX-2 enzyme.^33^ Optical imaging (Xenogen IVIS 200) at 49 h postinjection detected a high fluorescence in the tumor, suggesting the uptake of FQ-CA-NPs and ROS-mediated release of FQ and CA in the tumor followed by fluorescence activation of FQ (nonfluorescent) to FQ-H (fluorescent) (Figure 3b,c). The fluorescence signal was lowered significantly in the mammary tumor of the animal predosed with 10 mg/kg (i.p.) of tempol or celecoxib, followed by FQ-CA-NP injection. Tempol pretreatment traps tumor ROS to prevent cargo release from the loaded NPs and celecoxib pretreatment prevents binding of both FQ or CA with COX-2 by its active site blockade. After imaging, the animals were euthanized and the tumor, brain, liver, lung, and kidney were collected and imaged using a Xenogen IVIS 200 camera to measure the fluorescence intensity in these tissues. The measurement showed a significantly higher tumor signal in FQ-CA-NP animals compared to tempol- or celecoxib-pretreated animal tumors (Figure 3d,e).

To confirm the uptake of FQ-CA-NPs, the tumor tissues were analyzed by LC–MS/MS. The bioanalytical assay quantified the amount of FQ (0.115 nmol/g tissue) and CA (2.38 nmol/g tissue) delivered, released, and retained in the tumor, whereas a reduced amount of cargos was detected in the tumors of animals pretreated with tempol (FQ 8.32 pmol/g tissue and CA 11.87 pmol/g tissue) or pretreated with celecoxib (FQ 4.96 pmol/g tissue and CA 7.62 pmol/g tissue). No cargos were detected in untreated control tumors or tumors treated with empty-NPs. These fluorescence imaging and bioanalytical data confirmed the delivery of FQ-CA-NPs followed by FQ-mediated visualization of CA delivery in the mammary tumor and convincingly demonstrated the efficacy and specificity of FQ-CA-NPs for ROS and COX-2-expressing tumors.

**Figure 4:**
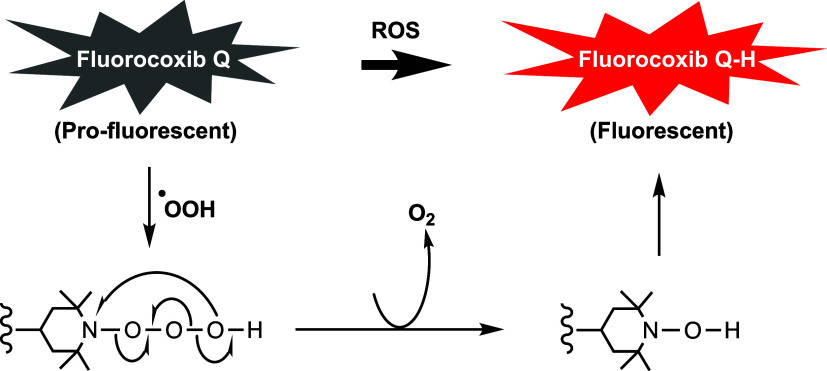
Proposed mechanism of ROS-mediated activation of FQ to highly fluorescent FQ-H.

The COX-2 enzyme has both an oxygenase activity that makes a peroxide product and a peroxidase activity that uses that product to generate an oxidant.^34,35^ COX-2 expression introduces oxidative stress in pathologic cells to cause DNA damage.^36^ Also, oxidative stress enhances the expression of NF-kappaB that induces COX-2 in pathological tissues.^37^ Thus, ROS and COX-2 colocalize in pathologic cells.^13^ It is possible that the nitroxide radical of the nonfluorescent FQ reacts with a peroxyl radical present in the tumor to afford an unstable 2,2,6,6-tetramethyl-1-trioxidaneylpiperidine intermediate in which the N–O bond session releases the 2,2,6,6-tetramethylpiperidyl radical, which traps a hydroxyl radical to afford the fluorescent FQ-H. This reaction liberates molecular oxygen as a byproduct of the redox reaction *in vivo* (Figure 4).

Polymer micellar nanoparticles release drugs through ROS-mediated oxidative degradation of their core. This drug release mechanism involves a phase change in the drug-loaded hydrophobic poly(propylene sulfide) (PPS) core, transforming it into a more hydrophilic form known as poly(propylene sulfoxide).^38^ Ultimately, this further degrades into the more water-soluble poly(propylene sulfone), releasing the hydrophobic drug from the oxidized nanoparticle core. The FQ-CA-NPs constitute an intelligent response system, which was oxidized by tumor ROS colocalized with COX-2. The ROS-induced disintegration of micellar FQ-CA-NPs allowed the release of individual drugs (FQ and CA) into the tumor microenvironment. In fact, the hydrophobic PPS unit in the micelles reacted with ROS to become hydrophilic, which induced the loaded NPs to rupture and allowed drug release. We performed two *in vivo* experiments to describe the mechanism of drug release of FQ-CA-NPs and subsequent fluorescence activation of pro-fluorescent FQ. One used tempol, an ROS-trapping agent, and the other used celecoxib, a selective COX-2 inhibitor. Each of these compounds was used 1 h prior to FQ-CA-NP injection. These pretreatments diminished the tumor signal significantly, confirming the ROS- and COX-2-dependent mechanism of *in vivo* and *ex vivo* visualization of drug delivery in breast tumors.

Finally, we tested the therapeutic efficacy of FQ-CA-NPs in a mouse model of triple-negative breast cancer. Mice bearing orthotopic MDA-MB-231 xenograft tumors were treated with four total doses of FQ-CA-NPs or empty-NPs (1 mg/kg drug content per dose, two doses per week). Treatment with FQ-CA-NPs resulted in significantly less tumor growth compared to treatment with empty-NPs (Figure S1). Furthermore, we determined that tumors treated with FQ-CA-NPs showed a statistically significant increase in the necrotic area on hematoxylin and eosin (H&E)-stained cross sections compared to those treated with empty-NPs (cross-sectional images are shown in Figure S2A,B, while quantification is shown in Figure S2C). Since increases in the size of the necrotic core are not necessarily reflected as a decrease in total tumor volume, this finding suggests a further therapeutic impact beyond what was measured in the tumor growth curves. Since the size of the necrotic core of a tumor can be related to the total tumor size, we ensured that the cross-sectional areas of the tumor sections selected for analysis were similar between the two groups (Figure S2D). Lastly, we performed H&E staining of healthy organs (liver, kidney, and spleen) from mice treated with FQ-CA-NPs or empty-NPs, which did not show any signs of toxicity from either treatment (Figure S2E).

These studies showed the feasibility of *in vivo* targeting of COX-2 in tumors using coloaded micellar nanoparticles to display the delivery of loaded compounds into breast tumors in live animals. In the presence of ROS in the breast tumors, the micellar nanoparticles (FQ-CA-NPs) were destabilized such that release of both cytotoxic agent CA and activatable probe FQ in tumor cells can take place to bind with intracellular COX-2 for enhanced tumor retention, where the activatable COX-2 probe FQ became fluorescently activated by tumor ROS allowing visualization of tumor delivery of cytotoxic agent in real time. Fluorescence activation of FQ was possible by interaction with ROS colocalized with COX-2 at the tumor site.^13^ The tumor ROS was blocked by tempol, and COX-2 was blocked by celecoxib, confirming the ROS- and COX-2-dependent mechanism of targeted cargo (FQ and CA) delivery and fluorescence activation of FQ in the tumor microenvironment. In addition, the CA-induced therapeutic effect was evaluated by an *in vivo* tumor growth inhibition assay and an *ex vivo* histopathological staining of tumor tissues. These experimental results suggest that the tumor uptake of FQ-CA-NPs followed by ROS-induced nanoparticle disassembly and cargo release for activation of FQ into the fluorescent species FQ-H for COX-2 binding to produce a detectable buildup of fluorescence signal required longer time (49 h) as compared to the free FQ compound (24 h).^13^

## Conclusions

We report the discovery of FQ-CA-NPs, a coloaded nanomedicine formulation based on polymeric micellar nanoparticles of FQ and CA. The FQ-CA-NPs showed sensitivity to ROS. 4T1 cells treated with FQ-CA-NPs showed higher fluorescence compared to celecoxib-pretreated 4T1 cells. FQ-CA-NPs showed toxicity to 4T1 breast carcinoma cells but were nontoxic to primary mammary cells. Following systemic administration, FQ-CA-NPs enabled the codelivery of FQ and CA in orthotopic mammary tumors. ROS-dependent cargo release followed by fluorescence activation of FQ in the tumor microenvironment allowed COX-2 binding for tumor accumulation and retention and detection of CA delivery into solid mammary tumors. The targeted codelivery of FQ and CA was confirmed by LC–MS/MS analysis of tumor tissues, which was lowered significantly in animals pretreated with either an ROS-trapping agent (tempol) or a COX-2 inhibitor (celecoxib). By combining disease diagnosis and therapy in one medical specimen, this study resulted in a new nanoplatform, where targeted tumor codelivery of an imaging and a therapeutic agent has been achieved.

## Supporting Information Available

Reagents and solvents, chromatography, and detailed reaction schemes for the chemical synthesis of fluorocoxib Q and chemocoxib A, as well as the synthesis of diblock copolymer; spectroscopic characterization of both small molecules and polymers, including ^1^H NMR spectra for all compounds, GPC of polymers, and HPLC chromatogram of fluorocoxib Q and chemocoxib A; synthetic methods and experimental details; preclinical optical imaging system for optical imaging of orthotopic breast tumors, tumor growth curves, and H&E-stained tumor cross sections providing comparisons of fluorescence Intensities, measurement of fluorescence intensity, and measurement of signal-to-noise ratio; and details and analytical techniques employed in the study (PDF)
